# The Mediating and Moderating Effects of Physical Fitness of the Relationship between Adherence to the Mediterranean Diet and Health-Related Quality of Life in University Students

**DOI:** 10.3390/nu12113578

**Published:** 2020-11-22

**Authors:** Noelia María Martín-Espinosa, Miriam Garrido-Miguel, Vicente Martínez-Vizcaíno, Alberto González-García, Andrés Redondo-Tébar, Ana Isabel Cobo-Cuenca

**Affiliations:** 1 Faculty of Physiotherapy and Nursing, Universidad de Castilla-La Mancha, 45071 Toledo, Spain; noelia.martin@uclm.es (N.M.M.-E.); anaisabel.cobo@uclm.es (A.I.C.-C.); 2 Health and Social Research Center, Universidad de Castilla-La Mancha, 16071 Cuenca, Spain; vicente.martinez@uclm.es (V.M.-V.); albertogonzalez@ugr.es (A.G.-G.); andres.redondo@uclm.es (A.R.-T.); 3 Faculty of Nursing, Universidad de Castilla-La Mancha, 02006 Albacete, Spain; 4 Facultad de Ciencias de la Salud, Universidad Autónoma de Chile, 1101 Talca, Chile; 5 Faculty of Health Sciences, Universidad de Granada, 18071 Granada, Spain; 6 Grupo de Investigación Multidisciplinaren Cuidados (IMCU), Universidad de Castilla-La Mancha, 45071 Toledo, Spain

**Keywords:** Mediterranean diet, health-related quality of life, fitness, muscle strength, young adults

## Abstract

The aim of this study was to estimate the relationship between the adherence to the Mediterranean diet (MD) and health-related quality of life (HRQoL) in university students and to assess whether this relationship is mediated or moderated by cardiorespiratory fitness (CRF) and handgrip strength. A cross-sectional study was performed involving 310 first-year Spanish university students. Adherence to the MD was evaluated with the 14-item Mediterranean Diet Adherence Screener (MEDAS), and the HRQoL was evaluated with the Short Form-12 (SF-12) questionnaire. CRF was assessed by the 20 m shuttle run test, and the handgrip strength was determined by dynamometry. ANCOVA models showed that participants with higher CRF and handgrip strength levels had significantly higher scores in the physical component summary (PCS) and mental component summary (MCS) of the SF-12 and in the MEDAS questionnaire than those with medium and low scores (*p* < 0.050). Additionally, the ANCOVA models showed that students with good adherence to the MD showed higher scores in the MCS of HRQoL than those with low adherence (*p* = 0.044, ES = 0.013), but these results did not appear for the PCS of HRQoL (*p* = 0.728, ES = 0.001). In the mediation analysis, it was found that CRF and handgrip strength acted as full mediators of the relationship between adherence to the MD and the MCS of HRQoL. In the moderation analysis, it was evidenced that CRF and handgrip strength did not act as moderators in the relationship between adherence to the MD and the MCS of HRQoL. In conclusion, adherence to the MD does not seem to have a direct effect on the MCS of HRQoL because this association seems to be fully mediated by CRF and handgrip strength.

## 1. Introduction

The interest in research of health-related quality of life (HRQoL) and its association with healthy lifestyles has grown in recent years; although, this subject has been less studied in young adults [1,2,3] than in other age groups [4,5,6,7,8]. HRQoL can be defined as a multidimensional concept that expresses a person’s self-perceived health state and that consists of several dimensions (physical, mental, emotional, behavioral, and social) of wellbeing and functionality [9]. Numerous studies on the instruments that measure HRQoL have recognized the existence of two main summaries [10,11]: the physical component summary (PCS) and the mental component summary (MCS).

The Mediterranean diet (MD) has been acknowledged as a healthy dietary pattern typical of inhabitants of countries surrounding the Mediterranean sea, and it is based on the high intake of legumes, vegetables, fruit, nuts, beans, fish, whole grains, unsaturated fats (olive oil), and the low consumption of dairy products and red meat [12]. A positive association between the adherence to the MD and HRQoL in both healthy adults [6,7] and adolescents [13,14] has been reported

Physical fitness is widely considered an important health marker through one’s lifespan, and it is related with a reduced incidence of cardiovascular disease and, consequently, its influence on improved survival rates has been confirmed [15,16,17]. The components of physical fitness associated to health include cardiorespiratory fitness (CRF) and muscle strength. Recently, research has demonstrated the impact of high levels of CRF and muscle strength on better health outcomes [18,19]. Furthermore, numerous studies have described that optimal levels of physical fitness prevent the onset of symptoms associated with depression and anxiety and improve the physical well-being of children and adolescents [20,21]. Furthermore, some research has analyzed the relationship between physical fitness and HRQoL in adolescents and young adults [2,3,14,22].

The association between adherence to the MD and physical fitness, and its influence on cardiovascular risk has been of recent interest to researchers [23,24]. Some current studies conducted in children and adolescents have stated that those with higher adherence to the MD presented the highest levels of physical fitness indicators such as CRF [25,26], muscle strength [25], and speed-agility [27]. The premises in which these relationships are based include that the high levels of antioxidants characteristics of the MD would theoretically lead to an increased efficiency in oxygen uptake and utilization. Futhermore, they may offer to protection of cellular components, such as proteins, from the catabolic effects of oxidative stress that imbalance the relationship between the production of reactive oxygen species and antioxidant defense [28]. Additionally, the combined associations between adherence to the MD and physical fitness with HRQoL have been scarcely studied in young adults. As far as we know, no studies have explored the associations between adherence to the MD combined with physical fitness and the improvement of the HRQoL in university students.

Thus, the aims of this study were to analyze the relationship between adherence to the MD and HRQoL and to test whether this relationship is mediated or moderated by different components of physical fitness (CRF and handgrip strength) in university students.

## 2. Materials and Methods 

### 2.1. Study Design and Participants

This was a cross-sectional study including university students enrolled in the first year at the University of Castilla-La Mancha (UCLM) during the 2017–2018 academic year.

A total of 510 students aged 18–30 years were invited to participate in the study, of which, 360 (64.3%) participated. The final sample included 310 first-year university students with a complete dataset, of which, women accounted for 65.16%. To ensure the results, missing data were imputed in a sensitivity analysis. The sex distribution was similar to that of the whole university campus population. 

The Clinical Research Ethics Committee of the “Virgen de la Luz” of Cuenca approved this study, which also complied with the principles of the Declaration of Helsinki (REG: 2016jPI1116). This article is part of the research “Lifestyle, adiposity and vascular function in college students from Castilla-La Mancha, Spain.” All participants read and signed the inform consent prior to their participation in the study.

### 2.2. Sample Size

The sample size was calculated by means of the software Epidat, estimating a prevalence of obesity of 23%, an alpha error of 0.05, statistical power of 80%, and a precision of 5% [29]. Estimating a rate of no response of 20%, the total size of the sample was 300 students. Taking as a sample frame the list of enrolments of these university courses, a random 560 students were invited, from which, 360 students agreed to participate. To determine the sample size, we have considered the obesity prevalence as outcome variable, since this study is part of the research “Lifestyle, adiposity and vascular function in college students from Castilla-La Mancha, Spain.” 

### 2.3. Study Variables

#### 2.3.1. Adherence to the Mediterranean Diet

The Mediterranean Diet Adherence Screener (MEDAS) [30] is a 14-item questionnaire that has been validated with Spanish people in which each item is scored 0 or 1, and the final score is the sum of each (0–14). Furthermore, this questionnaire has been used in samples of young adults [31,32,33]. Scores higher than 9 indicate good adherence to the MD. The first 12 questions refer to the frequency of food consumption (fruits, vegetables, olive oil, animal fats, red meat, fish/seafood, nuts, commercial foods, carbonated beverages, red wine, traditional dishes [with garlic, onion, tomato sauce, etc.]), and the two final questions are about cooking-fat preferences and meat consumed. Correlations between the MEDAS questionnaire and nutrient intake reported on the Food-Frequency Questionnaire (FFQ) (r = 0.52; intraclass correlation coefficient = 0.51) [34] and cardiovascular risk variables indicate a reasonable construct validity of the screener [30].

The FFQ [34] was used to determinate the total intake of carbohydrates, fats, proteins, and energy intake. This validated questionnaire contains 137 items with 9 levels of intake frequencies (never or almost never, 1–3 times per month, once per week, 2–4 times per week, 5–6 times per week, once per day, 2–3 times per day, 4–6 times per day, and more than 6 times per day). Energy and nutrient intakes were computed by using Spanish food composition tables [35].

#### 2.3.2. Health-Related Quality of Life

The Spanish Short Form-12 (SF-12) questionnaire [36] includes 12 items measuring 8 dimensions (physical function, physical role, body pain, general health, vitality, social function, emotional role, and mental health) that are usually grouped into 2 components: the PCS and the MCS. Higher scores indicate better physical and mental HRQoL, but, depending on age and sex, there are cut-off scores that need to be interpreted [37].

#### 2.3.3. Anthropometric Variables

Height was measured twice using a stadiometer SECA Model 222; Vogel & Halke; Hamburg, Germany; precision, 0.1 cm; range, 6–230 cm) and weight was defined by the average of two measurements obtained with an electronic scale (SECA Model 861; Vogel & Halke; Hamburg, Germany; precision, 100 g; range, 0–150 kg). Waist circumference was determined by the average of three measurements taken with flexible tape at the waist. To measure the waist circumference (cm), the researchers measured the midpoint between the iliac crest and the costal margin upon final exhalation. The mean of two measurements of weight and height was used to determine the body mass index (BMI) (weight [kg]/height [m^2^]). BMI was categorized into four groups: underweight (BMI ≤ 18.4), normal weight (18.5 ≤ BMI ≤ 24.9), overweight (25 ≤ BMI ≤ 29.9), and obese (BMI ≥ 30) [38]. Data collection was performed by trained nurses to reduce interobserver variability. Dual-energy X-ray absorptiometry (DEXA) (Lunar iDXA, GE Medical Systems Lunar, Madison, WI 53718, USA) was used to obtain the total fat mass (kg) and the total lean mass (kg). Two trained researchers performed all scans with high resolution, and the participants were placed in the supine decubital position.

#### 2.3.4. Physical Fitness

##### Handgrip Strength

The handgrip strength was used to measure the participant’s maximum handgrip force using a dynamometer (TKK 5401 Grip-D, Takeya, Tokyo, Japan). The test was performed twice with the right hand and twice with the left hand; the mean average of the 4 measurements was calculated. The standing long jump was used to measure lower explosive body strength. Participants stood behind a line with their feet approximately shoulder width apart and jumped as far as possible with both feet. The test measures the distance in centimeters from the starting line to the back of the participant’s heels. The best of 3 trials was recorded. Lastly, with the data of the two strength tests, a muscular strength index that consisted of the sum of the standardized z-scores of handgrip/weight and standing long jump was calculated.

##### Cardiorespiratory Fitness

The course navette test (20 m SRT) was used to assess the CRF. All students ran between 2 lines separated by 20 m. They had to keep up with the audio signals produced by a pre-recorded compact disc. In this test, the speed increased by 0.5 km/h every minute, with an initial speed of 8.5 km/h. Students were stimulated to keep running as long as possible during the course. The last stage completed was recorded. Leger’s formula was used to obtain estimates of submaximum oxygen consumption (VO_2_ max) [31.025 + (3.238 × velocity) − (3.248 × age) + (0.1536 × age × velocity)] [39].

#### 2.3.5. Family Socioeconomic Status

Data for the familiar socioeconomic level (SES) were gathered using self-reported occupation and education questions answered by both the father and mother. An index of SES was calculated according to the Spanish Society of Epidemiology scale procedures [40].

#### 2.3.6. Statistical Analysis

The Student’s *t*-test (continuous variables) or chi-squared test (categorical variables) were used to analyze the descriptive characteristics of the study sample by sex. The Kolmogorov–Smirnov test and graphical methods (normal probability plots) were used to check the normal distribution of continuous variables. All variables fitted acceptably to a normal distribution.

The Pearson correlation coefficient was used to determine the relationship between HRQoL domains (PCS, MCS) and body composition variables, CRF, handgrip strength, physical activity (PA), total energy intake (EI), and total MD scores.

CRF and handgrip strength were categorized as low (first quartile), medium (second and third quartile), and high (fourth quartile). The MEDAS questionnaire was categorized as low adherence (total score < 9) and good adherence (total score ≥ 9).

ANCOVA models were estimated to test the mean differences in HRQoL for CRF and handgrip strength and adherence to the MD categories. We also used ANCOVA models to test the differences in the mean of PCS and MCS by MEDAS-14 score categories, controlling for age, sex, and SES (model 1), and adding CRF as covariate (model 2) and handgrip strength (model 3). Pairwise post hoc hypotheses were tested using the Bonferroni correction for multiple comparisons. The size of the effect was categorized as small (0.01), moderate (0.06), or large (0.14) as classified by Cohen, 1988.

We carried out a mediation analysis to determine if CRF and handgrip strength were mediators in the relationship between the total MEDAS score and the MSC using the PROCESS macro for SPSS (SPSS Inc, Chicago, IL, USA).

Two strategies were used for this analysis: (1) a non-parametric strategy using a resample procedure of 10,000 bootstrap samples, as recommended Preacher and Hayes [41] and (2) a parametric strategy using the steps regression method, as recommended by Baron and Kenny [42]. The goal of this model was to investigate the total (c) and direct effects (a, b, c’), which indicates the unstandardized regression coefficient and significance between the independent and dependent variables in each model. It also investigates the indirect effect (IE), obtained from the product of coefficients (a * b), which shows the change in MCS for every unit change in the total score MEDAS that is mediated by physical fitness. Point estimates and confidence intervals (95%) were estimated for the confidence interval. The point estimate was considered to be significant when the confidence interval did not contain zero. The Sobel test was used [43] to test the statistical significance of the mediation effect in the parametric approach.

Additionally, the PROCESS macro for SPSS statistical software package, was used to conduct a moderation analysis. Moderation analysis was conducted to determine whether the relationship between total MEDAS and MCS was moderated by CRF and handgrip strength. This relationship used ordinary least squares regression analysis when predicting continuous variables (total MEDAS and physical fitness). A simple slope plot was used to visualize the effect of the moderator (Appendix A) [44].

SPSS-IBM (V.24.0 SPSS Inc., Armonk, NY, USA) was used to perform the statistical analyses, and the level of significance was set at *p* ≤ 0.05.

## 3. Results

A total of 310 students (108, 35.5% men) participated in the study. Table 1 shows the descriptive characteristics (mean ± standard deviation (SD)) of the study sample by sex. There were significant differences in weight, height, waist circumference, percentage of fat mass, total lean mass, CRF, muscle strength, and MSC between sexes.

Table 2 shows the bivariate correlations between HRQoL domains (PCS and MCS) with body composition, CRF, muscle strength, total EI, and total MEDAS score. The MCS, PCS, and total MEDAS score were positively associated with all body composition and physical fitness variables. Similarly, total MEDAS scores were also positively associated with the MCS of HRQoL.

Table 3 (Model 0) shows that the participants categorized with high CRF levels had significantly higher scores in the PCS and MCS of HRQoL than participants with medium and low scores (*p* < 0.001). Those categorized with high handgrip strength had significantly higher scores in the MCS than those with medium and low scores (model 0, *p* < 0.001; model 1, *p* = 0.045). In addition, those with high CRF and handgrip strength levels had higher scores on total score MEDAS (*p* < 0.05 and *p* = 0.04, respectively). When we adjusted for age, sex, and SES, these differences were maintained (Model 1).

Table 4 (Model 0) shows the mean-adjusted differences in the PCS and MCS according to the total MEDAS score (categorized as good adherence and low adherence) after controlling for potential confounders. The students with good adherence to the MD showed higher scores in the MCS than peers with low adherence, after controlling for age and sex (Model 1). Nevertheless, these differences disappeared when adjusting for CRF and handgrip strength (Model 2 and Model 3, respectively). There were no significant differences by MEDAS categories in PCS (*p* = 0.810).

### 3.1. Mediation Analysis

**Figure 1:**
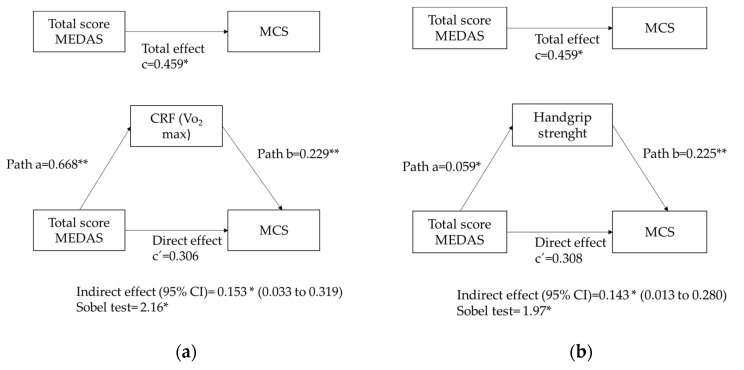
(**a**) Cardiorespiratory fitness CRF (VO_2_ max estimate) and (**b**) handgrip strength mediation models of the relationship between the Mediterranean Diet Adherence Screener (MEDAS) and mental HRQoL (MCS), controlling for age, sex, and socioeconomic status(SES). * *p* ≤ 0.05; ** *p* < 0.001.

Because the correlation coefficients and ANCOVA models did not indicate any relationship between the MEDAS score and the PCS, we only tested the potential mediator role of physical fitness between the MEDAS score and the MCS. In Figure 1a, the mediation analysis showed that the influence of the MEDAS score was mediated by CRF. Thus, the first regression equation showed that the relationship between the total MEDAS score and CRF was positive (a = 0.668, *p* < 0.001). In the second regression equation, the relationship between CRF and the MCS was positive (b = 0.229, *p* < 0.001). In the third regression equation, the relationship between the total MEDAS score and the MCS was positive (c = 0.459, *p* ˂ 0.05). Nevertheless, the relationship between the total MEDAS score and the MCS was attenuated when the mediator (CRF) was included in the regression (c´ = 0.306, *p* > 0.05). Therefore, CRF acted as a total mediator of the relationship between the MEDAS score and the MCS, according to the Sobel test = 2.16 (*p* < 0.001). Similar results were described when we tested the role of handgrip strength in the relationship between the total MEDAS score and the MCS (Figure 1b).

### 3.2. Moderation Analysis

Appendix A shows the results from the regression model, where it shows the moderation analysis based on ordinary least squares regression, in which no significant total score MEDAS × CRF/handgrip strength interaction effect on patients’ MCS score was found. The coefficient of moderation was not significant (a-CRF, B = 2.01, 95% CI, [−0.04, 1.93]; b-handgrip strength, B = 0.39, 95% CI, [−1.15, 1.93]), indicating that physical fitness (CRF/handgrip strength) did not moderate the relationship between adherence to the MD and MCS for young adults.

### 3.3. Sensitive Analysis

Assuming the data were missing at random, we conducted sensitivity analyses to test the robustness of the results with a multiple imputation techniques. A Markov chain Monte Carlo procedure was conducted with 20 iterations that included all the covariates, as well as independent and dependent variables included in the ANCOVA models. When the data were imputed, the results were similar and the statistical power increased (Tables S1 and S2 and Figure S1). 

## 4. Discussion

To the best of our knowledge, this is the first study to analyze the role of physical fitness in the relationship between adherence to the MD and the MCS of HRQoL in a sample of Spanish university students using a mediation and moderation analysis. Our data support that: (i) students with high adherence to the MD have higher values in the MCS of HRQoL; (ii) physical fitness is associated with different domains of HRQoL (PCS and MCS); (iii) CRF and handgrip strength act as total mediators of the relationship between adherence to the MD (total MEDAS score) and the MCS of HRQoL. 

The positive relationship between healthy diets and HRQoL in children and adolescents has been widely demonstrated in Spain [45,46] and in other countries [47,48,49]. In a recent systematic review, the intake of fast food, sweets, carbonated beverages, and salty snacks was associated with a low quality of life, whereas intake of yogurt, fruit, vegetables, and fish was associated with a better quality of life in the general population of children and adolescents [50].

In our study, good adherence to the MD was associated with better scores in MCS of HRQoL. It could be hypothesized that the lack of association with the PCS of HRQoL may be due to the age of the participants, young people whose health is commonly self-perceived as good. However, other studies carried out on mature adults [6] and elderly people [5] have found a positive association between adherence to the MD and the physical and mental components of HRQoL in both healthy and sick populations. In line with our findings, research has mostly reported a significant positive relationship between good adherence to the MD and the MCS of HRQoL [51,52]. Moreover, some studies have confirmed an inverse association between good quality diets and the risk of depression in both children [53,54] and adults [55,56,57,58]. Some biological processes related to the higher content of omega-3 polyunsaturated fatty acids in the MD (i.e., fish consumption) have been used to explain the beneficial effect of this dietary pattern in the functioning of the central nervous system through their potential interaction with both serotoninergic and dopaminergic transmission. Moreover, the high content of B vitamins in the MD seems to play a crucial role in some methylation reactions, which are implied in the synthesis of neurotransmitters, like serotonin [59]. Antioxidant nutrients can also improve the levels of serotonin, dopamine, and glutathione, preventing oxidative damage in the central nervous system [60].

The women in our sample have a lower mental HRQoL than men, which coincides with similar studies conducted in adolescents [61,62,63] and university students [3]. A study of adolescents aged 8 to 18 years showed that women had lower mHRQoL than men, also this difference increased with age [62]. This could be due to the fact that gender can influence the adoption of different lifestyles such as less vigorous practice exercises, gender role expectations, insecurities, among others. Furthermore, this difference between sexes could be due to social or gender factors and negative body image in girls [61].

In our sample, there was a positive significant association between physical fitness and the PCS and MCS of HRQoL. This is in line with other studies performed with children [20], adolescents [22], university students [2,3], and young adults [64]. In a sample of 1129 Norwegian children aged 10 years, Andersen et al. [65] determined that CRF had the strongest association with all domains of HRQoL. The importance of CRF in relation to mental health has been previously reported. According to a recent systematic review and meta-analysis, low and medium CRF levels are associated with a higher risk of developing common mental health conditions [66]. Other research has shown a higher prevalence of psychological distress or mental health problems in children, adolescents [67], and youngers with low physical fitness or PA levels [68]. 

In our research, high handgrip strength levels were associated with a better MCS of HRQoL. Some studies have shown that handgrip strength can be considered a good indicator of the PCS and MCS of HRQoL [69,70,71,72]. In line with our results, Kang et al. found that women with low handgrip strength were significantly more often depressed and anxious [72]. However, most research has found a positive relationship between handgrip strength and both dimensions of HRQoL in different population groups [22,73,74]. In line with our results, another study carried out with elderly people showed that high levels of handgrip strength were associated with better psychological functioning and sleep quality [71].

Using mediation analysis, our study supports the hypothesis that CRF and handgrip strength act as full mediators of the association between the MEDAS score and the mental dimension of HRQoL. To our knowledge, this is the first research that has reported this result. However, some preceding studies have stated that different components of physical fitness can act as mediators of HRQoL. For example, the role of CRF as a total mediator of HRQoL was previously described in a recent study carried out in Portuguese adolescents [14]. Another study of overweight and obese children reported that CRF and agility mediated the improvement of some HRQoL dimensions (academic functioning and physical, psychosocial, and total health) [75]. The positive effect of physical fitness on the promotion of several aspects of mental health is not well understood. It has been suggested that some diverse and complex biological mechanisms can be involved in this beneficial outcome. It seems that physical fitness optimizes physiological and neuroendocrine responses, inducing anti-inflammatory activity, insulin sensitivity, and neuroplasticity [76]. Further research is needed to clarify the potential factors behind the role of physical fitness on the MCS of HRQoL.

However, since high levels of physical fitness and adherence to the MD could have a beneficial effect on people’s health [33,77], it seems plausible that both may contribute to prevent the risk of some chronic diseases [61,78] and mental disorders [64,66,79,80,81]. Promoting activities that improve both parameters in young adults may be crucial to avoiding several health problems in adulthood and may enhance their quality of life.

Our study has some limitations that should be stated. First, the design of this study (cross-sectional study) does not allow for cause–effect relationships. Second, these results cannot be extrapolated to the overall population because the sample included only university students. Third, responses to self-completed questionnaires may be influenced by social desirability. Fourth, the study did not collect information on physical activity levels and sedentary behaviors. Fifth, this study was conducted in three provinces of Spain; thus, inferences to the whole Spanish population should be cautiously made. Further research with other population-based samples, additional variables, and a longitudinal study design would help to elucidate the relationship between adherence to the MD, physical fitness, and HRQoL.

## 5. Conclusions

Our data are relevant from a clinical perspective because they disclose that physical fitness variables play a pivotal role in the relationship between adherence to MD and mental dimension of HRQoL. Adherence to MD, per se, does not seem to have a direct effect because its association with HRQoL seems to be mediated by CRF and handgrip strength in young adults.

## Figures and Tables

**Table 1:** Descriptive characteristics of the study sample by sex.

	All (*n* = 310)	Men (*n* = 108)	Women (*n* = 202)	*p* *
Age (years)	20.9 ± 2.5	21.1 ± 2.8	20.7 ± 2.3	0.146
Weight (Kg)	65.4 ± 12.3	72.6 ± 10.99	61.4 ± 11.1	**<0.001**
Height (cm)	167.3 ± 8.6	175.3 ± 7.0	162.9 ± 5.8	**<0.001**
Waist circumference (cm)	78.9 ± 9.3	83.0 ± 7.9	76.6 ± 9.2	**<0.001**
% Fat mass	29.3 ± 9.0	20.5 ± 6.3	33.7 ± 6.7	**<0.001**
Total lean mass (Kg)	43.0 ± 9.3	53.5 ± 6.8	37.8 ± 4.9	**<** **0.001**
BMI (Kg/m^2^)	23.3 ± 3.5	23.5 ± 3.03	23.1 ± 3.8	0.269
Underweight (%)	3.1	0.8	4.4	
Normal weight (%)	70.6	70.6	70.6	0.068
Overweight (%)	21.8	26.2	19.3	
Obesity (%)	4.5	2.4	5.7	
CRF (stages)	5.8 ± 2.6	7.0 ± 1.9	3.8 ± 1.4	**<0.001**
CRF (VO_2_ max estimate, ml/Kg/min)	37.5 ± 8.0	44.4 ± 6.6	32.8 ± 4.9	**<0.001**
Muscle strength index (cm/Kg) ^a^	0.013 ± 1.7	1.523 ± 1.2	−1.050 ± 1.2	**<0.001**
Handgrip strength (Kg)	30.4 ± 9.5	39.2 ± 7.7	24.4 ± 4.7	**<0.001**
Standing long jump (cm)	161.2 ± 43.7	195.5 ± 31.9	136.8 ± 33.5	**<0.001**
EI (Kcal)	2795.7 ± 1804.7	2865.9 ± 1287.0	2757.6 ± 2033.2	0.590
Carbohydrate (% EI)	43.0 ± 7.1	43.1 ± 6.6	42.9 ± 7.3	0.852
Protein (% EI)	17.4 ± 3.4	17.4 ± 3.2	17.5 ± 3.6	0.749
Fat (% EI)	38.2 ± 6.2	37.9 ± 5.9	38.3 ± 6.3	0.578
Health-related quality of life ^b^ (SF-12)				
PCS	54.7 ± 5.5	54.7 ± 5.3	54.6 ± 5.6	0.827
MCS	40.0 ± 6.4	42.1 ± 5.9	38.6 ± 6.4	**<0.001**
Adherence Mediterranean Diet (%)				
Low adherence	65.4	70.4	79.0	0.090
Good adherence	24.0	29.6	21.0	
Total score MEDAS	7.0 ± 2.0	7.2 ± 1.9	6.9 ± 2.0	0.214

Results are shown as mean and ± SD. For categorical variables, the values are expressed in percentages. Bold values indicate statistical significance *p* ≤ 0.05. Abbreviations: BMI body mass index; CRF, cardiorespiratory fitness; EI, energy intake; MEDAS, Mediterranean Diet Adherence Screener; PCS, physical component summary; MCS, mental component summary. ^a^ Sum of the standardized z score of dynamometry/weight and standing long jump. ^b^ Higher scores indicate a better health-related quality of life. * T student tests (continuous variables), or chi squared tests (categorical variables).

**Table 2:** Bivariate correlations between health-related quality of life (HRQoL) domains with body composition, cardiorespiratory fitness (CRF), muscle strength, physical activity (PA), total energy intake (EI), and total Mediterranean Diet Adherence Screener (MEDAS-14) score.

	PCS	MCS	BMI	WC	% Fat Mass	Total Lean Mass	CRF (Steges)	CRF (VO_2_ Max Estimate)	Handgrip Strength	Total EI	Total Score MEDAS
**PCS**	-	−0.446 *	−0.240 **	−0.223 **	−0.252 **	−0.014	0.124	0.129 *	−0.102	0.018	0.023
**MCS**		-	0.075	0.156 **	−0.030	0.272 **	0.279 **	0.272 **	0.335 **	0.044	0.160 **
**BMI**			-	0.802 **	0.493 **	0.337 **	−0.188 **	−0.190 **	0.224 **	−0.116 *	0.164 **
**WC**				-	0.217 *	0.544 *	−0.007	−0.010	0.383 **	−0.057	0.130 *
**% Fat mass**					-	−0.496 **	−0.548 **	−0.540 **	−0.390 **	−0.166 **	0.016
**Total lean mass**						-	0.628 **	0.621 **	0.780 **	−0.061	0.144 *
**CRF (stages)**							-	0.996 **	0.597 **	0.188 **	0.155 *
**CRF (VO_2_ max estimate)**								-	0.589 **	0.176 **	0.155 *
**Handgrip strength**									-	0.101	0.139 *
**Total EI**										-	0.088

Data are presented in the correlation coefficient R. * *p* < 0.05, ** *p* < 0.001. Abbreviations: BMI, body mass index; EI, energy intake; HRQoL, health-related quality of life; PCS, physical component summary; MCS, mental component summary; WC, waist circumference.

**Table 3:** ANCOVA models comparing the means of physical HRQoL (PCS), mental HRQoL (MCS), and total Mediterranean Diet Adherence Screener (MEDAS-14) score according to categories of cardiorespiratory fitness (CRF) and handgrip strength.

	CRF (VO_2_ Max Estimate, mL/Kg/min)					Handgrip Strength (Kg)				
	Low	Medium	High	*p*	ES ^d^	Low	Medium	High	*p*	ES ^d^
*n*	60	121	66			65	132	63		
**PCS**										
Model 0	53.5 ± 6.9 ^a^	55.7 ± 5.2	55.6 ± 4.6	**0.031**	0.03	56.1 ± 4.7	55.1 ± 5.9	54.5 ± 5.3	0.251	0.01
Model 1	52.8 ± 6.0 ^a,c^	55.5 ± 5.2	56.6 ± 4.5	**0.007**	0.04	56.5 ± 4.3	55.1 ± 5.9	53.9 ± 5.2	0.157	0.01
**MCS**										
Model 0	38.1± 7.1 ^a,c^	38.7 ± 6.3 ^b^	42.8 ± 6.1	**<0.001**	0.08	37.5 ± 6.8 ^a^	39.0 ± 6.1 ^b^	42.9 ± 6.3	**<0.001**	0.09
Model 1	38.57 ± 7.2	38.98 ± 6.3 ^b^	42.0 ± 6.2	**0.044**	0.02	38.2 ± 6.8	39.2 ± 6.2	41.8 ± 6.4	0.055	0.02
**Total MEDAS**										
Model 0	6.7 ± 2.0 ^a^	6.9 ± 2.1	7.6 ± 2.1	**0.050**	0.03	6.3 ± 1.9	7.0 ± 2.1	7.5 ± 2.2	**0.040**	0.03
Model 1	6.5 ± 2.0 ^a^	6.8 ± 2.0	7.7 ± 2.1	0.067	0.03	6.3 ± 2.0 ^a^	7.1 ± 2.1	7.5 ± 2.2	0.052	0.02

Values are marginal estimated means ± SD. Bold values indicate statistical significance *p* ≤ 0.05. Abbreviation: CRF, cardiorespiratory fitness; ES; effect size (partial eta-squared); PCS, physical component summary; MCS, mental component summary. Categories of CRF, and handgrip strength are: low (representing 1st quartile), medium (2nd and 3rd quartiles), and high (4th quartile). Superscript letters indicate statistical significance (*p* < 0.05) in pairwise mean comparisons using Bonferroni post-hoc test: ^a^ low < high, ^b^ medium < high, ^c^ low < medium. Model 0 Crude data; Model 1 adjusted for age + sex + SES. ^d^ The size of the effect was categorized as small (0.01), moderate (0.06) or large (0.14) as classified by Cohen, 1988.

**Table 4:** ANCOVA models comparing the means of physical HRQoL(PCS) and mental HRQoL(MCS) with the Mediterranean Diet Adherence Screener (MEDAS-14) items categories after controlling for cardiorespiratory fitness (CRF) and handgrip strength.

	Adherence to the MD			
	Low Adherence	Good Adherence	*p*	ES ^a^
*n*	232	74		
**PCS**				
Model 0	54.5 ± 5.4	54.4 ± 5.1	0.728	0.001
Model 1	54.7 ± 5.0	54.5 ± 5.0	0.819	0.001
Model 2	55.3 ± 5.6	55.8 ± 5.1	0.573	0.001
Model 3	55.2 ± 4.9	55.4 ± 5.0	0.683	0.001
**MCS**				
Model 0	39.6 ± 6.7	41.4 ± 5.7	**0.044**	0.013
Model 1	39.5 ± 6.6	41.1 ± 5.8	0.095	0.009
Model 2	39.3 ± 6.8	40.9 ± 6.0	0.113	0.011
Model 3	39.2 ± 5.9	40.9 ± 6.8	0.089	0.012

Values are marginal estimated means ± SD. Bold values indicate statistical significance *p* ≤ 0.05. Abbreviations: ES; effect size (partial eta-squared); PCS, physical component summary; MCS, mental component summary. Low adherence = total score < 9 on the MEDAS-14 items questionnaire; good adherence = total score ≥ 9 on the MEDAS-14 items questionnaire. Model 0: Crude data; Model 1: Age + sex + SES; Model 2: Model 1+ CRF; Model 3: Model 1+ handgrip strength. ^a^ The size of the effect was categorized as small (0.01), moderate (0.06) or large (0.14) as classified by Cohen, 1988.

**Figure A1:**
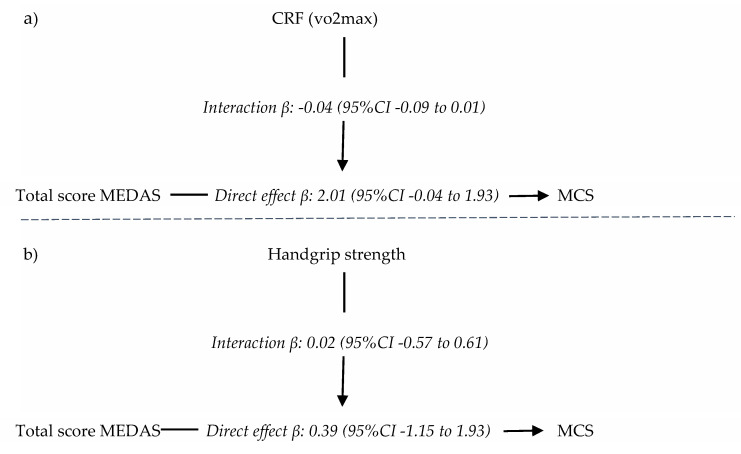
(**a**) CRF VO_2_ max estimate and (**b**) handgrip strength moderation models of the relationship between the total MEDAS score and mental HRQoL (MCS). Adjusted by age, sex, and SES. *Beta* expressed as unstandardized regression coefficients and 95% confidence interval.

## Supplementary Materials

The following are available online at https://www.mdpi.com/2072-6643/12/11/3578/s1, Figure S1: (A) CRF Vo2 max estimate and (B) handgrip strength mediation models of the relationship between the total MEDAS score and mental HRQoL (MCS). * *p* ≤ 0.05; ** *p* < 0.001, Table S1: ANCOVA models comparing the means of physical HRQoL (PCS), mental HRQoL (MCS), and total MEDAS-14 scores according to categories of CRF and handgrip strength, Table S2: ANCOVA models comparing the means of the PCS and the MCS with the MEDAS-14 items categories after controlling for CRF and handgrip strength.
