# Supplementary material for: The Mediating and Moderating Effects of Physical Fitness of the Relationship between Adherence to the Mediterranean Diet and Health-Related Quality of Life in University Students
Source: Nutrients. 2020 Nov 22;12(11):3578. doi: 10.3390/nu12113578 (PMC7700278; doi:10.3390/nu12113578)
Supplement: Supplementary file 1 [file nutrients-12-03578-s001.pdf]

Supplementary material

Table S1. ANCOVA models comparing the means of physical HRQoL (PCS), mental HRQoL (MCS), and total MEDAS-14 scores according to categories of CRF and handgrip strength.

	**CRF (VO_2 max estimate, ml/Kg/min)**					**Handgrip strength (Kg)**				
	Low	*Medium*	*High*	*P*	*ES* [d]	*Low*	*Medium*	*High*	*p*	*ES* [d]
**n**	83	195	82			88	181	91		
**PCS**										
Model 0	53.3±6.6[a]	55.0±5.2	55.3±4.6	**0.005**	0.02	55.1±4.7	54.4±5.9	54.4±5.3	0.458	0.004
Model 1	52.8±6.0[a,c]	55.0±5.2	55.7±4.5	**0.004**	0.03	55.5±4.3	54.5±5.9	54.1±5.2	0.317	0.001
**MCS**										
Model 0	38.6± 7.1 [c]	39.1±6.1[b]	42.7±5.6	**<0.001**	0.08	38.1±6.8[a]	39.3±6.1[b]	42.9±6.3	**<0.001**	0.08
Model 1	39.2±7.2[c]	39.3±6.3[b]	41.7±6.2	**0.044**	0.01	38.7±6.8	39.4±6.2	41.2±6.4	**0.040**	0.02
**Total MEDAS**										
Model 0	6.7±2.0[a]	6.9±2.1	7.5±2.1	**0.033**	0.02	6.7±1.9[a]	7.0±2.1	7.5±2.2	**0.034**	0.02
Model 1	6.7±2.0	6.8±2.0	7.5±2.1	0.078	0.01	6.7±2.0[a]	6.9±2.1	7.5±2.2	0.158	0.01

Values are marginal estimated means ± SD. Bold values indicate statistical significance $p \leq 0.05$. Abbreviation: CRF, cardiorespiratory fitness; ES; effect size (partial eta-squared); PCS, physical component summary; MCS, mental component summary. Categories of CRF, and handgrip strength are: Low (representing 1st quartile), Medium (2nd and 3rd quartiles), and high (4th quartile). Superscript letters indicate statistical significance (P < 0.05) in pairwise mean comparisons using Bonferroni post-hoc test: [a]low < high, [b] medium<high, [c]low < medium. Model 0 Crude data; Model 1 Adjusted for age + sex + socioeconomic level. [d]The size of the effect was categorized as small (0.01), moderate (0.06) or large (0.14) as classified by Cohen, 1988.

Table S2. ANCOVA models comparing the means of the PCS and the MCS with the MEDAS-14 items categories after controlling for CRF and handgrip strength.

	**Adherence to the MD**			
	Low adherence	*Good adherence*	*p*	*ES*[a]
n	234	126		
**PCS**				
Model 0	54.7±5.4	54.6±5.1	0.870	0.001
Model 1	54.7±5.0	54.6±5.0	0.944	0.001
Model 2	55.3±5.6	55.7±5.1	0.669	0.001
Model 3	55.1±4.9	55.3±5.0	0.714	0.001
**MCS**				
Model 0	39.2±6.7	41.3±5.7	**0.006**	0.021
Model 1	39.5±6.6	40.9±5.8	**0.031**	0.013
Model 2	39.3±6.8	40.9±6.0	0.054	0.011
Model 3	39.3±5.9	40.9±6.8	0.087	0.012

Values are marginal estimated means ± SD. Bold values indicate statistical significance P≤0.05. Abbreviations: ES; effect size (partial eta-squared); PCS, physical component summary; MCS, mental component summary.Low adherence=total score < 9 on the MEDAS-14 items questionnaire; good adherence= total score ≥ 9 on the MEDAS-14 items questionnaire.Model 0: Crude data; Model 1: Age + sex + socioeconomic level. ; Model 2: Model 1+ CRF; Model 3: Model 1+ handgrip strength. [a]The size of the effect was categorized as small (0.01), moderate (0.06) or large (0.14) as classified by Cohen, 1988.

A) **B)**

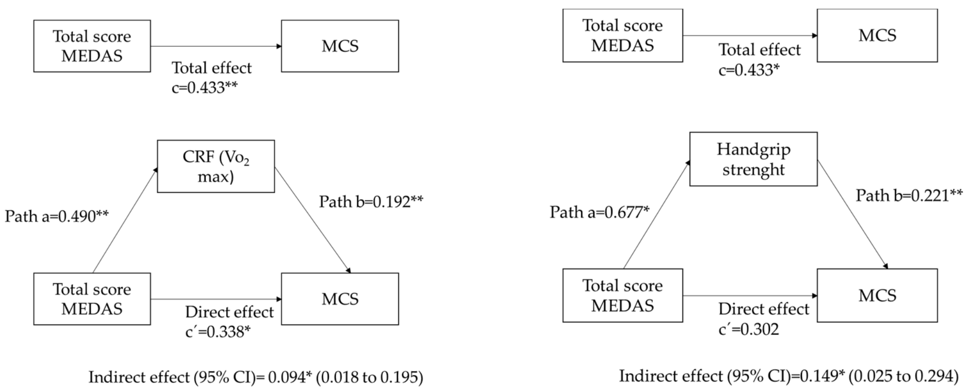

Figure S2. A) CRF Vo2 max estimate and B) handgrip strength mediation models of the relationship between the total MEDAS score and mental HRQoL (MCS). *p ≤ 0.05; **p < 0.001.
